# Radiographic prevalence and associated factors of hallux rigidus in a large-scale population-based cohort

**DOI:** 10.1016/j.ocarto.2025.100695

**Published:** 2025-10-11

**Authors:** Yuko Yagi, Takumi Matsumoto, Akihiro Uchio, Ryutaro Takeda, Toshiko Iidaka, Chiaki Horii, Hiroyuki Oka, Shigeyuki Muraki, Hiroshi Hashizume, Hiroshi Yamada, Munehito Yoshida, Kozo Nakamura, Sakae Tanaka, Noriko Yoshimura

**Affiliations:** a Department of Orthopaedic Surgery, Otakanomori Hospital, Japan; b Department of Orthopaedic Surgery, Faculty of Medicine, The University of Tokyo, Japan; c Department of Preventive Medicine for Locomotive Organ Disorders, 22nd Century Medical & Research Center, Faculty of Medicine, University of Tokyo, Tokyo, Japan; d Division of Musculoskeletal AI System Development, Faculty of Medicine, The University of Tokyo, Japan; e Muraki Orthopedic Clinic, Japan; f School of Health and Nursing Science, Wakayama Medical University, Wakayama, Japan; g Department of Orthopaedic Surgery, Wakayama Medical University, Wakayama, Japan; h Department of Orthopaedic Surgery, Sumiya Orthopedic Hospital, 337 Yoshida, Wakayama City, Wakayama, Japan; i Towa Hospital, Tokyo, Japan

**Keywords:** Hallux rigidus, Osteoarthritis, Foot joints, Gout, Risk factors

## Abstract

**Objective:**

Hallux rigidus (HR), a form of osteoarthritis (OA) affecting the first metatarsophalangeal joint, significantly impairs mobility and quality of life. Despite its clinical importance, large-scale epidemiological studies on radiographic HR prevalence and associated factors remain limited. This study investigated the radiographic prevalence of HR and its associated factors in a large, population-based Japanese cohort.

**Design:**

Data were obtained from 1998 participants (654 men, 1344 women) in the fifth survey of the nationwide Research on Osteoarthritis/Osteoporosis Against Disability (ROAD) study. Non-weight-bearing dorsoplantar foot radiographs were assessed using the Hattrup and Johnson classification (grade 1: mild, grade 2: moderate, and grade 3: severe); HR was defined as grade ≥1. Multivariable logistic regression assessed associated factors, and the Cochran-Armitage trend test evaluated severity-related trends.

**Results:**

The mean age was 64.2 ​± ​12.7 years. The overall HR prevalence was 23.5 ​% (25.1 ​% in men, 22.8 ​% in women), with no significant sex difference. Unilateral and bilateral HR were 6.6 ​% and 17.0 ​% of participants, respectively. HR was classified as mild (13.3 ​%), moderate (7.0 ​%), or severe (3.3 ​%). Multivariable analysis identified older age, coastal residence, gout history, and knee OA as independent factors associated with HR. HR severity showed significant linear trends with both gout and knee OA.

**Conclusions:**

This study determined the radiographic prevalence of HR of 23.5 ​% in a Japanese population. Independent factors associated with HR included older age, coastal residence, gout, and knee OA. Findings indicate that HR is multifactorial and highlight the need to explore geographic and lifestyle-related factors in addition to medical conditions.

## 1 Introduction

Hallux rigidus (HR) is a form of osteoarthritis (OA) that affects the first metatarsophalangeal (1st MTP) joint [1]. Among the joints of the foot, the 1st MTP joint is the most common site of OA [2,3]. As its name implies, hallux rigidus is characterized by a limited range of motion, particularly restricted dorsiflexion [4]. When symptomatic, it is often associated with pain, swelling, and functional impairment, leading to reduced mobility, difficulty in finding appropriate footwear, and decreased foot-specific and physical quality of life [5].

The etiology of HR is considered multifactorial, with contributing factors including advanced age, female sex, prior trauma, familial predisposition, racial background, gout, poorly fitting footwear, hind foot valgus, and structural abnormalities of the first ray [3,4,[6], [7], [8], [9], [10]]. Despite the clinical significance of HR, large-scale epidemiological studies assessing its prevalence and associated factors remain limited, particularly those based on general populations. For example, a study in the United Kingdom (UK) estimated the prevalence of symptomatic HR at 7.8 ​%, where foot radiographs were obtained only from participants who reported foot pain during the last 12 months, with radiographic assessment based on the La Trobe atlas [3]. Although this provides valuable information on the prevalence of symptomatic HR, radiographic changes and clinical symptoms do not always correspond, and the prevalence of radiographic HR in general population samples, irrespective of symptoms, remains unclear [11,12].

A population-based radiographic survey conducted in the Netherlands evaluated OA at 22 joint sites in over 6000 community-dwelling adults, using the Kellgren–Lawrence grading system, and reported that approximately 20 ​% of individuals already had radiographic OA of the 1st MTP joint by age 40 ^2^. In another community-based cohort of Caucasian and African American adults in the United States, the radiographic prevalence of 1st MTP joint OA was 10.4 ​%, evaluated with the La Trobe atlas [13]. However, these studies addressed OA at multiple joint sites in a general framework, rather than specifically analyzing HR, and did not include severity grading or detailed assessment of associated risk factors at the first MTP joint. A longitudinal cohort study in the UK reported that the prevalence of radiographic HR, based on the La Trobe atlas, was 33.2 ​% at baseline and increased to 40.9 ​% over 19 years among 193 females; however, the study included only females and had a relatively small sample size [14]. In Japan, a community-based study conducted among 604 individuals aged over 50 reported a radiographic prevalence of 26.7 ​%, classified according to the Hattrup and Johnson system, and identified several associated factors, including knee OA and gout [9]. However, its limited sample size and geographic restriction to a single rural area may limit generalizability.

Therefore, the aim of the present study was to investigate the radiographic prevalence of HR and its associated factors in a large, population-based cohort.

## 2 Method

### 2.1 Study design

Data for the present analysis were obtained from the fifth survey of the Research on Osteoarthritis/Osteoporosis Against Disability (ROAD) study, a nationwide, population-based cohort launched in 2005 to investigate musculoskeletal conditions in Japan. The ROAD study comprises participants from three regions in Japan: an urban area (Itabashi, Tokyo), a mountainous area (Hidakagawa, Wakayama), and a coastal area (Taiji, Wakayama). Details regarding the study's design and data collection methods have been reported previously [15,16].

The fifth survey, conducted between 2018 and 2019 in the mountainous and coastal areas, enrolled a total of 2386 participants (943 from the mountainous area and 1443 from the coastal area). Participants were eligible if they were able to walk to the clinic and complete self-administered questionnaires. Among the 2386 participants, 1998 individuals (654 males and 1344 females) with non-weight-bearing dorsoplantar radiographs available for both feet were included in the analysis. Other than missing radiographs, no specific exclusion criteria were applied.

From the data collected in the ROAD study, information relevant to the present study was extracted, including participant demographics, laboratory test results, and self-administered questionnaires addressing medical history and medication use. Medical history was based on self-reported physician-diagnosed conditions such as hypertension, dyslipidemia, gout, and cardiovascular diseases.

The study was conducted in accordance with the Declaration of Helsinki and was approved by the institutional ethics committee. Written informed consent was obtained from all participants prior to enrollment.

### 2.2 Radiographic assessment

**Fig. 1:**
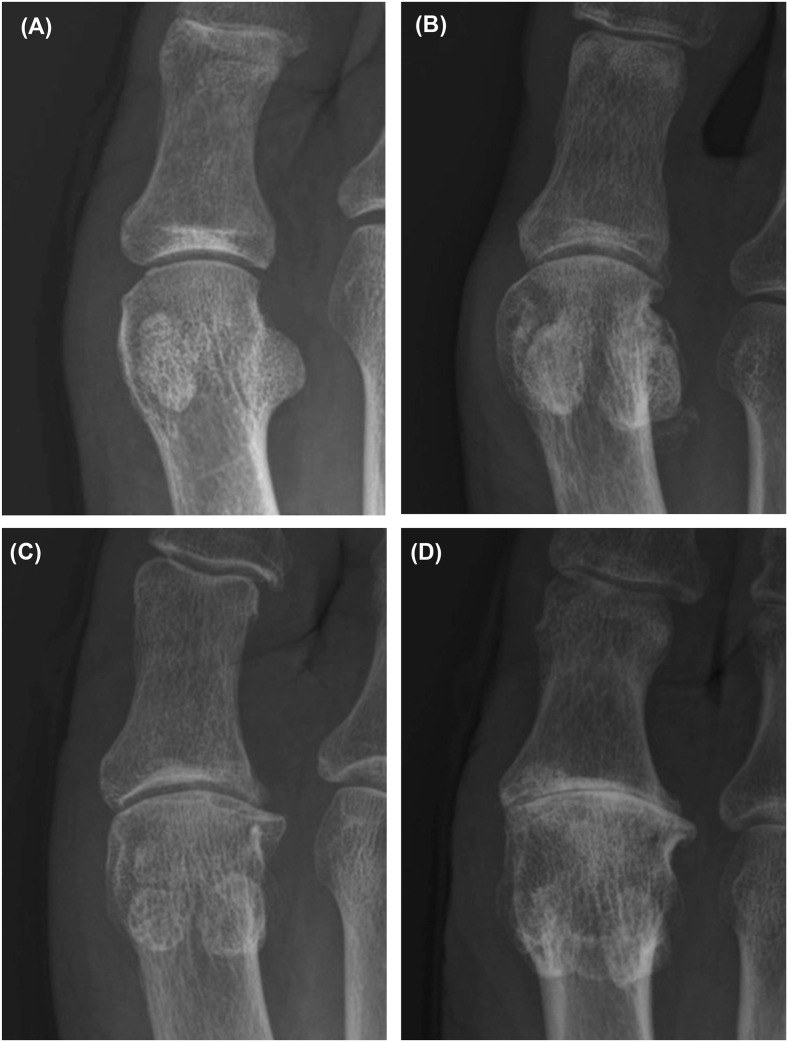
Representative foot radiographs illustrating the Hattrup and Johnson classification of hallux rigidus severity: (**A**) grade 0, normal; (**B**) grade 1, mild to moderate osteophytes without joint space narrowing (JSN); (**C**) grade 2, moderate osteophytes with less than 50 ​% JSN and subchondral sclerosis; and (**D**) grade 3, marked osteophytes with 50 ​% or more JSN.

Non-weight-bearing dorsoplantar radiographs of both feet and weight-bearing anteroposterior radiographs of both knees were obtained. HR was graded according to the Hattrup and Johnson classification as follows: grade 0, normal; grade 1, mild to moderate osteophytes without joint space narrowing (JSN); grade 2, moderate osteophytes with less than 50 ​% JSN and subchondral sclerosis; and grade 3, marked osteophytes with 50 ​% or more JSN (Fig. 1) [7,17]. When radiographic findings did not align neatly with the Hattrup and Johnson categories, greater weight was placed on osteophytes (spurs), in line with evidence that osteophytes alone identify the majority of OA cases [18]. Radiographic HR was defined as grade 1 or higher according to this classification.

The severity of radiographic knee OA was assessed using the Kellgren-Lawrence (KL) classification system, which classifies OA severity as follows: grade 0, no radiographic abnormalities; grade 1, minor osteophytes; grade 2, definite osteophytes; grade 3, JSN accompanied by multiple osteophytes; and grade 4, bone sclerosis along with JSN and large osteophytes [19]. Participants with a history of total knee arthroplasty were classified as having KL grade 4 OA. Radiographic knee OA was defined as a KL grade of 2 or greater.

All radiographic images were assessed by a single experienced orthopedic surgeon specializing in foot and ankle surgery, who was blinded to the participants’ clinical information to ensure unbiased and standardized evaluations.

To assess intra-rater reliability of the Hattrup and Johnson classification in our cohort, we conducted an additional reproducibility analysis. A random sample of 100 feet was selected from the study population. Radiographs were independently re-evaluated by the same experienced foot and ankle surgeon after a washout period of more than three months, blinded to the initial scores. Agreement between the two readings was quantified using Cohen's kappa coefficients: unweighted, linear weighted, and quadratic weighted κ, with the latter considered most appropriate for ordinal data. The reproducibility was excellent, with a quadratic weighted κ of 0.91, a linear weighted κ of 0.82, and an unweighted κ of 0.69. Discrepancies mainly occurred between adjacent grades (e.g., grade 1 vs grade 2).

### 2.3 Statistics

Statistical summary results are presented as means with standard deviations (SDs) or as counts and percentages. Group comparisons were performed at the individual level, based on the presence or absence of HR in either foot, using the chi-square test or Fisher's exact test, as appropriate. The Mann-Whitney *U* test was used for continuous variables.

A multivariable logistic regression analysis was performed with the presence of HR as the dependent variable. Independent variables included basic characteristics (sex, age, body mass index [BMI], and residential area), as well as comorbidities−including radiographic knee OA−that showed significant associations in univariate analyses. For this analysis, age was categorized into quartiles, and BMI was classified as underweight (<18.5 ​kg/m^2^), normal weight (18.5–24.9 ​kg/m^2^), or overweight (≥25.0 ​kg/m^2^). Odds ratios (ORs) with 95 ​% confidence intervals (CIs) and P-values were reported.

For analyses involving HR severity, each participant's severity was defined by the higher grade observed between the right and left feet. The Cochran-Armitage test for trend was used to assess the relationship between HR severity and the variables that showed significant associations with HR in the multivariable logistic regression analysis. In addition, a departure-from-trend test was conducted to evaluate whether each association followed a linear pattern across severity levels. A P-value <0.05 was considered statistically significant. All statistical analyses were performed using Stata version 17.0 (StataCorp LLC, College Station, TX, USA).

### 2.4 Ethics consideration

Ethical approval for the study was obtained from the ethics committee of the University of Tokyo (Approval No. 1264). This study was conducted in accordance with the principles of the Declaration of Helsinki, and written informed consent was obtained from all participants.

## 3 Results

The demographic and clinical characteristics of the participants are summarized in Table 1, while laboratory findings and medication use are presented separately in Supplementary Table 1. These variables were compared between the non-HR and HR groups for males and females separately. Among male participants, the presence of HR was significantly associated with older age, residence in the coastal area, radiographic knee OA, medical history of gout, angina, and myocardial infarction, and the use of urate-lowering medications and nonsteroidal anti-inflammatory drugs. Among female participants, HR was significantly associated with older age, greater height, higher BMI, residence in the coastal area, radiographic knee OA, medical history of hypertension and dyslipidemia, laboratory parameters (lower HDL cholesterol, higher triglycerides, and higher HbA1c according to the National Glycohemoglobin Standardization Program), and the use of antihypertensive, cholesterol-lowering, and antidiabetic medications.

**Table 1:** Comparison of participants’ characteristics between the non-HR and HR groups in males and females.

Variables	Total	Male					Female				
	(N ​= ​1998)	non-HR	HR	MD/OR	95%CI	P value	non-HR	HR	MD/OR	95%CI	P value
		(N ​= ​490)	(N ​= ​164)				(N ​= ​1038)	(N ​= ​206)			
Age (years)	64.2 ​± ​12.7	63.0 ​± ​13.9	66.4 ​± ​13.0	3.4	1.0 to 5.9	0.003^a^	63.4 ​± ​12.2	68.0 ​± ​11.0	4.6	3.2 to 6.2	<0.001^a^
Height (cm)	158.0 ​± ​9.1	167.2 ​± ​6.8	166.9 ​± ​6.4	−0.3	−1.5 to 0.8	0.55	153.8 ​± ​6.4	152.6 ​± ​6.2	−1.2	−2.0 to −0.4	<0.001^a^
Weight (kg)	56.9 ​± ​11.6	65.8 ​± ​11.0	66.2 ​± ​11.3	0.4	−1.5 to 2.4	0.84	52.4 ​± ​8.9	53.2 ​± ​9.2	0.8	−0.3 to 2.0	0.13
Body Mass Index (kg/m^2^)	22.7 ​± ​3.5	23.5 ​± ​3.2	23.8 ​± ​3.6	0.3	−0.3 to 0.9	0.53	22.2 ​± ​3.5	22.9 ​± ​3.6	0.7	0.3 to 1.1	<0.001^a^
Coastal residence (number, [%])	1169 (58.5 ​%)	270 (55.1 ​%)	107 (65.2 ​%)	1.53	1.06 to 2.21	0.02^a^	574 (55.3 ​%)	218 (71.2 ​%)	2.00	1.52 to 2.64	<0.001^a^
Radiographic knee OA (number, [%])	809 (40.5 ​%)	127 (25.9 ​%)	63 (38.4 ​%)	1.78	1.23 to 2.59	0.002^a^	446 (43.0 ​%)	174 (56.5 ​%)	1.73	1.33 to 2.23	<0.001^a^
Medical history (number, [%])											
Osteoporosis	241 (12.2 ​%)	13 (2.7 ​%)	4 (2.5 ​%)	0.92	0.30 to 2.86	>0.99	173 (16.8 ​%)	51 (17.0 ​%)	1.02	0.72 to 1.43	0.92
Hypertension	791 (39.6 ​%)	205 (41.8 ​%)	78 (47.6 ​%)	1.26	0.88 to 1.80	0.20	365 (35.2 ​%)	143 (46.7 ​%)	1.62	1.25 to 2.09	<0.001^a^
Dyslipidemia	604 (30.4 ​%)	130 (26.6 ​%)	43 (26.4 ​%)	0.99	0.66 to 1.48	0.95	308 (29.8 ​%)	123 (40.6 ​%)	1.61	1.24 to 2.10	<0.001^a^
Diabetes mellitus	190 (9.5 ​%)	73 (14.9 ​%)	26 (15.9 ​%)	1.08	0.66 to 1.75	0.77	64 (6.2 ​%)	27 (8.9 ​%)	1.48	0.93 to 2.37	0.10
Gout	125 (6.3 ​%)	73 (14.9 ​%)	38 (23.3 ​%)	1.74	1.12 to 2.70	0.01^a^	8 (0.8 ​%)	6 (2.0 ​%)	2.58	0.89 to 7.50	0.10
Angina	89 (4.5 ​%)	27 (5.5 ​%)	17 (10.4 ​%)	1.98	1.05 to 3.74	0.03^a^	30 (2.9 ​%)	15 (4.9 ​%)	1.73	0.92 to 3.26	0.09
Myocardial infarction	34 (1.7 ​%)	14 (2.9 ​%)	11 (6.7 ​%)	2.44	1.08 to 5.49	0.03^a^	6 (0.6 ​%)	3 (1.0 ​%)	1.71	0.42 to 6.87	0.43
Asthma	147 (7.4 ​%)	29 (5.9 ​%)	13 (8.0 ​%)	1.38	0.70 to 2.72	0.35	85 (8.2 ​%)	20 (6.5 ​%)	0.78	0.47 to 1.30	0.34
Depression	74 (3.7 ​%)	19 (3.9 ​%)	5 (3.1 ​%)	0.78	0.29 to 2.12	0.63	42 (4.1 ​%)	8 (2.6 ​%)	0.64	0.30 to 1.38	0.25
Gastric ulcer	251 (12.6 ​%)	97 (19.9 ​%)	30 (18.5 ​%)	0.92	0.58 to 1.44	0.71	100 (9.7 ​%)	24 (7.9 ​%)	0.80	0.50 to 1.28	0.35
Duodenal ulcer	131 (6.6 ​%)	39 (8.0 ​%)	19 (11.6 ​%)	1.51	0.84 to 2.69	0.16	50 (4.8 ​%)	23 (7.5 ​%)	1.60	0.96 to 2.67	0.07
*H. pylori* infection	447 (22.4 ​%)	116 (23.7 ​%)	39 (23.8 ​%)	1.01	0.66 to 1.52	0.97	220 (21.2 ​%)	72 (23.5 ​%)	1.14	0.84 to 1.55	0.69
History of gastric surgery	84 (4.2 ​%)	30 (6.1 ​%)	17 (10.4 ​%)	1.77	0.95 to 3.31	0.07	32 (3.1 ​%)	5 (1.6 ​%)	0.52	0.20 to 1.35	0.17
Dialysis	11 (0.6 ​%)	4 (0.8 ​%)	0 (0.0 ​%)	–	–	0.58	5 (0.5 ​%)	2 (0.7 ​%)	1.36	0.26 to 7.04	0.66
Cancer	229 (11.5 ​%)	66 (13.5 ​%)	21 (12.8 ​%)	0.94	0.56 to 1.60	0.83	102 (9.8 ​%)	40 (13.1 ​%)	1.38	0.93 to 2.04	0.11
Stroke	61 (3.1 ​%)	22 (4.5 ​%)	7 (4.3 ​%)	0.95	0.40 to 2.26	0.90	22 (2.1 ​%)	10 (3.3 ​%)	1.56	0.73 to 3.34	0.25
Rheumatoid arthritis	51 (2.6 ​%)	5 (1.0 ​%)	3 (1.8 ​%)	1.81	0.43 to 7.65	0.42	34 (3.3 ​%)	9 (3.0 ​%)	0.89	0.42 to 1.88	0.76
Parkinson's disease	6 (0.3 ​%)	3 (0.6 ​%)	0 (0.0 ​%)	–	–	0.58	2 (0.2 ​%)	1 (0.3 ​%)	1.69	0.15 to 18.75	0.54

Abbreviations: OA, osteoarthritis; *H. pylori*, *Helicobacter pylori*; HR, hallux rigidus; MD, mean difference; OR, odds ratio; CI, confidence interval.

Percentages were calculated using the number of participants with available data for each variable as the denominator. The total N varied slightly across variables due to missing responses. Effect measures are presented as MDs with 95 ​% CIs for continuous variables, ORs with 95 ​% CIs for categorical variables.

a Statistically significant difference between non-HR and HR groups, as determined by the chi-square test or Fisher's exact test for categorical variables, and the Mann–Whitney *U* test for continuous variables.

The proportions of participants with radiographic HR in either foot were 23.5 ​% among the 1998 individuals (25.1 ​% in males and 22.8 ​% in females), with no significant sex difference (P ​= ​0.25) (Table 2). Unilateral HR was observed in 6.6 ​% of participants (5.4 ​% in males and 7.1 ​% in females), while bilateral HR was found in 17.0 ​% (19.7 ​% in males and 15.6 ​% in females). Despite the overall prevalence showing no sex difference, the distribution of HR laterality differed significantly between male and female (P ​= ​0.03). Residual analysis indicated that bilateral HR was significantly more common in males (P ​= ​0.03), while no significant sex difference was observed for unilateral HR. Regarding severity, the prevalence of mild (grade 1), moderate (grade 2), and severe (grade 3) HR was 13.3 ​% (14.7 ​% in males and 12.6 ​% in females), 7.0 ​% (7.2 ​% in males and 6.9 ​% in females), and 3.3 ​% (3.2 ​% in males and 3.3 ​% in females), respectively. There was no significant difference in the distribution of HR severity between males and females (P ​= ​0.61).

**Table 2:** Radiographic prevalence of HR by sex and age quartile.

	Total	Male	Female
Age quartile			
Q1 (Age:21–55)	77/503 (15.3 ​%)	32/179 (17.9 ​%)	45/324 (13.9 ​%)
Q2 (Age:56–65)	110/553 (19.9 ​%)	38/170 (22.4 ​%)	72/383 (18.8 ​%)
Q3 (Age:66–72)	132/457 (28.9 ​%)	47/149 (31.5 ​%)	85/308 (27.6 ​%)
Q4 (Age:73–95)	151/485 (31.1 ​%)	47/156 (30.1 ​%)	104/329 (31.6 ​%)
Overall	470/1998 (23.5 ​%)	164/654 (25.1 ​%)	306/1398 (22.8 ​%)

Values are presented as n (%).

A multivariable logistic regression analysis was conducted with the presence of HR as the dependent variable. Independent variables included sex, age (categorized into quartiles), BMI (underweight, normal weight, and overweight), residential area (mountainous or coastal area), and comorbidities that were significantly associated with HR in univariate analyses (radiographic knee OA, history of hypertension, dyslipidemia, gout, angina, and myocardial infarction). Laboratory findings and medication use were excluded from the multivariable model due to their strong clinical correlation with existing comorbidities, which could lead to multicollinearity and compromise model interpretability. The analysis identified older age, coastal residence, radiographic knee OA, and history of gout as independent factors significantly associated with HR (Table 3). Compared with the youngest quartile (21–55 years), the ORs for HR were 2.11 (95 ​% CI: 1.50–2.97, P ​< ​0.001) in the third quartile (66–72 years) and 2.33 (95 ​% CI: 1.62–3.35, P ​< ​0.001) in the oldest quartile (73–95 years). Coastal residence was also associated with a significantly higher risk of HR compared to mountainous residence (OR: 2.09, 95 ​% CI: 1.66–2.63, P ​< ​0.001). Additionally, radiographic knee OA (OR: 1.29, 95 ​% CI: 1.01–1.64, P ​= ​0.04) and a history of gout (OR: 1.61, 95 ​% CI: 1.04–2.47, P ​= ​0.03) were identified as significant risk factors.

**Table 3:** Multivariable logistic regression analysis for factors associated with hallux rigidus.

	Odds ratio	95 ​% CI	P value
Female	0.93	0.73–1.20	0.59
Age quartile			
Q1 (Age:21–55)	(Reference)		
Q2 (Age:56–65)	1.38	0.99–1.93	0.06
Q3 (Age:66–72)	2.11	1.50–2.97	<0.001^a^
Q4 (Age:73–95)	2.33	1.62–3.35	<0.001^a^
Body Mass Index			
Underweight (<18.5 ​kg/m^2^)	(Reference)		
Normal (18.5–24.9 ​kg/m^2^)	1.23	0.81–1.87	0.34
Overweight (≥25.0 ​kg/m^2^)	1.5	0.94–2.40	0.09
Coastal residence	2.09	1.66–2.63	<0.001^a^
Radiographic knee OA	1.29	1.01–1.64	0.04^a^
Medical history			
Hypertension	1.04	0.81–1.33	0.78
Dyslipidemia	1.07	0.84–1.36	0.60
Gout	1.61	1.04–2.47	0.03^a^
Angina	1.28	0.77–2.11	0.34
Myocardial infarction	1.33	0.62–2.87	0.46

Abbreviations: OA, osteoarthritis; CI, confidence interval.

a Statistically significant association.

The Cochran-Armitage trend test demonstrated significant positive associations between HR severity and both a history of gout (Z ​= ​3.45, P ​< ​0.001) and radiographic knee OA (Z ​= ​6.31, P ​< ​0.001). For both variables, the test for departure from the linear trend was non-significant (P ​= ​0.74 and P ​= ​0.31, respectively), indicating a linear trend. In contrast, although HR severity was also significantly higher in the coastal area than in the mountainous area (Z ​= ​2.71, P ​= ​0.007), the departure-from-trend test suggested that this relationship did not follow a simple linear pattern (P ​< ​0.001).

## 4 Discussion

The present study investigated HR in a large, population-based cohort of 1998 community-dwelling Japanese adults, aiming to clarify its radiographic prevalence and associated factors in the general population. We found that approximately one in four participants had radiographic HR. This is one of the largest studies to date to examine HR in the general population. Multivariable analysis revealed that older age, residence in the coastal area, radiographic knee OA, and a history of gout were independently associated with its presence.

The overall radiographic prevalence of HR in this study was 23.5 ​%, including 13.3 ​% classified as mild, 7.0 ​% as moderate, and 3.3 ​% as severe. This finding aligns closely with a previous Japanese community-based study by Senga et al. (n ​= ​604), conducted in a single rural region, which reported a radiographic prevalence of 26.7 ​%, with a similar distribution of severity grades (16.4 ​% grade 1, 8.0 ​% grade 2, and 2.3 ​% grade 3) using the same Hattrup and Johnson classification as in the present study [9]. These similarities may be attributable to the use of radiographic criteria and comparable population characteristics within Japanese community cohorts. A similar prevalence has also been reported in a large-scale Dutch population-based radiographic survey, which included over 6000 community-dwelling adults [2]. In that study, OA was defined radiographically using the Kellgren–Lawrence grading system; they found that approximately 20 ​% of individuals already had radiographic OA of the 1st MTP joint by the age of 40. Although that study is one of the largest to evaluate OA across multiple joint sites, its relatively younger age distribution—more than half of the participants were aged 20 to 44 years—limits direct comparison. Additionally, a community-based cohort in the United States involving 848 adults also reported a relatively low radiographic prevalence of HR (10.4 ​%) among Caucasian and African American adults, assessed using the La Trobe atlas [13]. The relatively small sample size of that study, however, should be noted as a limitation. A longitudinal study in the UK documented a baseline radiographic HR prevalence of 33.2 ​% among 193 females, which subsequently rose to 40.9 ​% over a 19-year period, also using the La Trobe atlas; however, that study was limited by its small sample size and exclusive inclusion of female participants [14]. In addition, a UK-based radiographic study of 517 adult patients presenting to the emergency department with acute foot injuries found radiographic HR in 25 ​% of cases, graded according to the Hattrup and Johnson classification [20]. Although the mean age of that cohort was relatively young (47 years), the reported radiographic prevalence was comparable to that in our study. However, this finding may not be generalizable to the broader population, as the sample was limited to individuals with acute trauma. Another UK study involving community-dwelling adults aged ≥50 estimated the population prevalence of symptomatic radiographic HR at 7.8 ​%, with radiographs assessed using the La Trobe atlas and prevalence estimates calculated based on weighted analyses that accounted for missing data and non-response [3]. Importantly, radiographs in that study were obtained only from participants who had experienced foot pain within the past 12 months. As a result, the reported prevalence likely reflects only symptomatic cases.

Our findings demonstrated an age-related increase in the prevalence of radiographic HR, with significantly higher odds observed in the third (66–72 years) and fourth (73–95 years) age quartiles. Although not reaching statistical significance (P ​= ​0.06), the second age quartile (56–65 years) also showed a trend toward increased odds of HR (OR ​= ​1.38). This finding is consistent with a previous report, using the Hattrup and Johnson classification, showing that the radiographic prevalence of HR increases sharply with age, with relative risks approximately twofold after the age of 60 and threefold after 80 ^2,20^. Given that HR is a form of OA, its age-related increase is not unexpected [2,3,21]. In addition to local mechanical stress, systemic factors related to aging, such as low-grade inflammation, metabolic changes, and reduced regenerative capacity, may also contribute to the pathogenesis of HR [22].

In the present study, radiographic knee OA was significantly associated with HR (OR: 1.29, 95 ​% CI: 1.01–1.64), and participants with knee OA tended to show more severe HR, consistent with a dose-response relationship. This association corroborates previous findings indicating a link between radiographic HR and knee OA [9]. Additional evidence suggests that HR often co-occurs with OA at other joint sites. For example, a population-based study, using the Kellgren–Lawrence grading system, reported that individuals with knee OA had significantly higher odds of having radiographic HR (adjusted OR: 3.7, 95 ​% CI: 3.0–4.5), even after adjustment for age, BMI, and occupational history. A similar strong association was observed with OA in hand joints, such as the distal interphalangeal, proximal interphalangeal, and carpometacarpal joints (adjusted ORs: 3.2–3.6), when using the Coughlin and Shurnas radiographic classification system [21]. Moreover, radiographic HR was identified in 72.9 ​% of patients undergoing total ankle arthroplasty for end-stage ankle arthritis [23]. Although that study lacked a control group, the radiographic prevalence was substantially higher than in population-based cohorts. When stratified by the etiology of ankle arthritis, HR was more frequent in primary and inflammatory arthritis (OR: 1.18 and 1.31, respectively) than in post-traumatic arthritis, although these differences were not statistically significant. Taken together, the association between HR and OA at multiple joint sites suggests that systemic factors may contribute to the development and progression of HR, supporting the perspective that HR may be a part of a generalized osteoarthritic phenotype rather than an isolated joint disorder [24].

A further notable finding of the present study was the independent association between a history of gout and the presence of radiographic HR (OR: 1.61, 95 ​% CI: 1.04–2.47), with a significant linear trend observed across HR severity grades. This finding is consistent with previous studies reporting an association between gout and HR [9,25,26]. Notably, our study's larger sample size allowed for the demonstration of a significant linear trend with HR severity, providing more robust evidence of this relationship than previously available [9]. This discrepancy may be attributable to the smaller sample size in that study, which could have limited the statistical power to detect associations with disease severity. In contrast, the larger sample size in our study allowed for a more precise and robust estimation of the association. Some authors have suggested that the relationship between gout and HR may be confounded by shared metabolic risk factors such as obesity [27]. However, in our multivariable analysis, gout remained significantly associated with HR even after adjusting for BMI and other potential confounders, indicating an independent contribution of gout.

The present study revealed that the prevalence of radiographic HR was significantly higher in the coastal area than in the mountainous area (OR: 2.09, 95 ​% CI: 1.66–2.63), indicating a clear regional difference. To our knowledge, no previous studies have specifically examined geographic variation in HR prevalence within a single country. Evidence from international epidemiological studies on OA suggests that lifestyle and environmental factors may play a substantial role in shaping regional patterns. For instance, a population-based study in rural Jamaica, in which radiographs were graded using the “Standard Atlas of Radiographs”, observed a significantly lower prevalence of radiographic OA of the 1st MTP joint compared to a British agricultural cohort [28]. The authors speculated that this difference might be related to lifestyle and environmental factors, such as habitual barefoot walking. Similarly, in a rural South African population, radiographic OA of the 1st MTP joint—assessed using the Kellgren–Lawrence grading system— was observed in 15.1 ​% of males and 24.1 ​% of females, both significantly lower than in British populations [29]. Although dated, these studies are among the few to have explored international variation in 1st MTP joint OA, and their findings remain valuable for understanding potential environmental and cultural influences. These international findings, together with the regional disparity observed in our study, suggest that environmental, occupational, cultural, socioeconomic, or biomechanical factors may contribute to the development of HR. Although such factors have been proposed, they have not been systematically studied [4,6,7]. Moreover, these regional differences may also, in part, reflect differences in genetic or ethnic backgrounds, though such influences remain largely unexplored. Given potential variations in diagnostic criteria, imaging methods, and population characteristics, cross-study comparisons should be interpreted with caution. Although our study did not investigate the underlying environmental or lifestyle factors, the clear regional variation observed within a single country underscores the importance of future research to identify modifiable contributors to HR development.

In the present study, although the difference was not statistically significant, the radiographic prevalence of HR was slightly higher in males (25.1 ​%) than in females (22.8 ​%). This contrasts with most previous reports, which have suggested a female predominance, although some studies have indicated a higher prevalence in males [3,6,20,23]. The variation in sex-specific prevalence may reflect differences in the burden of gout, which is more common in males, as well as lifestyle-related factors such as mechanical loading and occupational exposure.

This study has several limitations. First, its cross-sectional design precludes any conclusions about causal relationships between HR and associated factors. Second, participants were recruited exclusively from rural communities, which may limit the generalizability of the findings to urban populations. Third, the diagnosis of HR was based solely on radiographic findings without incorporating clinical symptoms. Fourth, the radiographic evaluation of the 1st MTP joint was based only on non–weight-bearing anteroposterior views without lateral projections, which may have limited the detection of certain features such as dorsal osteophytes. In a previous radiographic study, the sensitivity of dorsoplantar view alone for detecting radiographic HR was reported to be 77.2 ​%, indicating that the addition of lateral views would be desirable in future investigations to improve diagnostic accuracy [18]. To our knowledge, no published data address potential differences in sensitivity between non-weight-bearing and weight-bearing radiographs for the evaluation of HR. However, increasing weight-bearing primarily alters hindfoot and midfoot alignment, whereas forefoot parameters remain unchanged between non-weight-bearing and weight-bearing conditions [30]. This finding suggests that the influence of non-weight-bearing positioning on HR assessment may be limited, although some degree of underestimation of prevalence cannot be excluded. Fifth, HR was defined using the Hattrup and Johnson classification. Although this scale has been widely used in both clinical and epidemiologic studies, including a large population-based study in Japan, there is currently no established gold standard for the radiographic classification of HR [9,31]. A report describing fair to good intra-rater reliability and excellent inter-rater reliability indicates that the use of this scale is reasonable in the context of our study [32]. Sixth, although information on rheumatoid arthritis was collected and showed no association with HR, other forms of inflammatory arthritis, such as psoriatic arthritis, were not specifically assessed and therefore could not be excluded. Given the very low prevalence of inflammatory arthritis in Japan, the potential impact on our prevalence estimates is likely negligible [33]. Seventh, the inclusion criterion requiring participants to attend the clinic physically may have introduced selection bias, potentially limiting the generalizability of our findings. Individuals with severe pain or significant functional impairment may have been unable to attend in person. Consequently, the study sample may underrepresent those with more advanced or symptomatic hallux rigidus, possibly leading to an underestimation of its true prevalence and severity in the general population. In addition, some information, including medical history, was self-reported and may be subject to recall bias. Finally, although several potential confounders were adjusted for, residual confounding cannot be ruled out.

This large-scale population-based study revealed that HR is relatively common in the Japanese community-dwelling population, with a radiographic prevalence of 23.5 ​%. Beyond known risk factors such as older age, gout, and knee OA, the observed regional variation in HR prevalence suggests that environmental or geographic factors may also contribute to its development. These findings highlight the multifactorial etiology of HR and underscore the importance of exploring not only medical but also geographic and lifestyle-related factors. Future studies including urban populations and longitudinal designs are needed to clarify the natural history of HR and to inform strategies for prevention and early intervention.

## Author contributions

Conception and design: TM, AU, RT, ST, NY.

Analysis and interpretation of the data: TM, RT, YY.

Drafting of the article: YY, TM.

Final approval of the article: All authors.

Provision of study materials or patients: TI, CH, HO, SM, NY.

Statistical expertise: HO.

Obtaining of funding: TI, HO, ST, NY.

Administrative, technical, or logistic support: TI, CH, HH, HY, MY, KN, ST, NY.

Collection and assembly of data: AU, RT, TI, CH, HH, HY, NY.

## Role of funding source

This work was supported by a Grant-in-Aid funding from the Ministry of Health, Labour and Welfare: H17-Men-eki-009 (Director, Kozo Nakamura), H23-Choujyu-002 (Director, Toru Akune), 19FA1014, 19FA1007 and 20JA1001 (Director, Hiroyuki Oka), 19FA1017 (Director, Estuo Chosa), 19FB1001 (Director, Yutaka Osuga), 21FA1006 (Director, Hiroshi Yamada), H25-Nanchitou (Men)-005, 22FA1009 and 24FA1003 (Director, Sakae Tanaka), H20-Choujyu-009, H25-Choujyu-007 and 24FA1006 (Director, Noriko Yoshimura). The study was also supported by Scientific Research grants B25K02882, B19H03895, B26293139, B23390172, and B20390182 and Challenging Exploratory Research grants 21K19631, 18K18447, 15K15219, and 24659317 to Noriko Yoshimura; Challenging Exploratory Research grants 21K18291 to Kanae Mure, Young Scientists 17H06628 and 23K16322 to Toshiko Iidaka; Scientific Research grants 18K10063, 22K10552, and 25K13603 to Izumi Inoue; Scientific Research grants B26293331, B23390356, and C20591774 and Challenging Exploratory Research grants 26670307 and 23659580 to Shigeyuki Muraki; Challenging Exploratory Research grants 24659666 and 21659349 and Young Scientists A18689031 to Hiroyuki Oka; Scientific Research grants B26293329, B23390357, and C20591737 and Challenging Exploratory Research grant 25670293 to Toru Akune; Scientific Research grant 19H05654 to Sakae Tanaka; and by Collaborating Research with NSF 08033011-00262 (Director, Noriko Yoshimura) from the Ministry of Education, Culture, Sports, Science and Technology in Japan). The study was partly supported by grants from the Japan Agency for Medical Research and Development (JP17dk0110028, Director, Noriko Yoshimura; JP15gk0210007, JP18gk0210018, JP22gk0210034, and JP25gk0210046, Director, Sakae Tanaka; JP22dk0110048, Director, Hiroyuki Oka; JP22dk0110047, Director, Kanae Mure; JP24rea522115, Director, Akiko Kishi; JP223fa627011, Director, Yuji Yamanashi. Further, the study was partly supported by grants from the Japan Osteoporosis Society (Noriko Yoshimura, Toshiko Iidaka, Shigeyuki Muraki, Hiroyuki Oka, and Toru Akune) and Japan Osteoporosis Foundation (2015, Noriko Yoshimura) and research aids from the Japanese Orthopaedic Association (JOA-Subsidized Science Project Research 2006-1 and 2010-2, Director, Hiroshi Kawaguchi; and 2014-1, Director, Kozo Nakamura), the Japanese Society for Musculoskeletal Medicine (2015, Director, Shigeyuki Muraki; and 2017, Director, Noriko Yoshimura), Mitsui Sumitomo Insurance Welfare Foundation (2016, Director, Noriko Yoshimura; and 2024, Director, Toshiko Iidaka), Grant-in-Aid for the Japan Hip Joint Foundation (2016, Director, Toshiko Iidaka), Nakatomi Foundation (2019, Director, Toshiko Iidaka), Japan Dairy Association (2017, Director, Noriko Yoshimura), and Suzuken Memorial Foundation (2023, Director, Noriko Yoshimura).

## Conflict of interest

All authors declare no conflict of interests and disclose no financial or personal relationships with other people or organizations that could inappropriately influence this work.

## References

[1] Cotterill J.M. (1887). Stiffness of the great toe in adolescents. Br Med J.

[2] van Saase J.L., van Romunde L.K., Cats A., Vandenbroucke J.P., Valkenburg H.A. (1989). Epidemiology of osteoarthritis: zoetermeer survey. Comparison of radiological osteoarthritis in a Dutch population with that in 10 other populations. Ann. Rheum. Dis..

[3] Roddy E., Thomas M.J., Marshall M., Rathod T., Myers H., Menz H.B. (2015). The population prevalence of symptomatic radiographic foot osteoarthritis in community-dwelling older adults: cross-sectional findings from the clinical assessment study of the foot. Ann. Rheum. Dis..

[4] Bingold A.C., Collins D.H. (1950). Hallux rigidus. J Bone Joint Surg Br.

[5] Bergin S.M., Munteanu S.E., Zammit G.V., Nikolopoulos N., Menz H.B. (2012). Impact of first metatarsophalangeal joint osteoarthritis on health-related quality of life. Arthritis Care Res..

[6] Gould N., Schneider W., Ashikaga T. (1980). Epidemiological survey of foot problems in the continental United States: 1978-1979. Foot Ankle.

[7] Coughlin M.J., Shurnas P.S. (2003). Hallux rigidus: demographics, etiology, and radiographic assessment. Foot Ankle Int..

[8] Zammit G.V., Menz H.B., Munteanu S.E. (2009). Structural factors associated with hallux limitus/rigidus: a systematic review of case control studies. J. Orthop. Sports Phys. Ther..

[9] Senga Y., Nishimura A., Ito N., Kitaura Y., Sudo A. (2021). Prevalence of and risk factors for hallux rigidus: a cross-sectional study in Japan. BMC Muscoskelet. Disord..

[10] Mahiquez M.Y., Wilder F.V., Stephens H.M. (2006). Positive hindfoot valgus and osteoarthritis of the first metatarsophalangeal joint. Foot Ankle Int..

[11] Lawrence J.S., Bremner J.M., Bier F. (1966). Osteo-arthrosis. Prevalence in the population and relationship between symptoms and x-ray changes. Ann. Rheum. Dis..

[12] Munteanu S.E., Zammit G.V., Menz H.B. (2012). Factors associated with foot pain severity and foot-related disability in individuals with first metatarsophalangeal joint OA. Rheumatology.

[13] Golightly Y.M., Hannan M.T., Nelson A.E., Hillstrom H.J., Cleveland R.J., Kraus V.B. (2019). Relationship of joint hypermobility with ankle and foot radiographic osteoarthritis and symptoms in a community-based cohort. Arthritis Care Res..

[14] Bowen C., Gates L., McQueen P., Daniels M., Delmestri A., Drechsler W. (2020). Natural history of radiographic first metatarsophalangeal joint osteoarthritis: a nineteen-year population-based cohort Study. Arthritis Care Res..

[15] Yoshimura N., Muraki S., Oka H., Mabuchi A., En-Yo Y., Yoshida M. (2009). Prevalence of knee osteoarthritis, lumbar spondylosis, and osteoporosis in Japanese men and women: the research on osteoarthritis/osteoporosis against disability study. J. Bone Miner. Metabol..

[16] Yoshimura N., Muraki S., Oka H., Kawaguchi H., Nakamura K., Akune T. (2010). Cohort profile: research on Osteoarthritis/Osteoporosis against Disability study. Int. J. Epidemiol..

[17] Hattrup S.J., Johnson K.A. (1988). Subjective results of hallux rigidus following treatment with cheilectomy. Clin. Orthop. Relat. Res..

[18] Menz H.B., Munteanu S.E., Marshall M., Thomas M.J., Rathod-Mistry T., Peat G.M. (2022). Identification of Radiographic Foot Osteoarthritis: sensitivity of Views and Features Using the La Trobe Radiographic Atlas. Arthritis Care Res..

[19] Kellgren J.H., Lawrence J.S. (1957). Radiological assessment of osteo-arthrosis. Ann. Rheum. Dis..

[20] Howard N., Cowen C., Caplan M., Platt S. (2014). Radiological prevalence of degenerative arthritis of the first metatarsophalangeal joint. Foot Ankle Int..

[21] Wilder F.V., Barrett J.P., Farina E.J. (2005). The association of radiographic foot osteoarthritis and radiographic osteoarthritis at other sites. Osteoarthr. Cartil..

[22] Loeser R.F., Collins J.A., Diekman B.O. (2016). Ageing and the pathogenesis of osteoarthritis. Nat. Rev. Rheumatol..

[23] Bejarano-Pineda L., Cody E.A., Nunley J.A. (2021). Prevalence of Hallux rigidus in patients with end-stage ankle arthritis. J. Foot Ankle Surg..

[24] Nelson A.E., Smith M.W., Golightly Y.M., Jordan J.M. (2014). "Generalized osteoarthritis": a systematic review. Semin. Arthritis Rheum..

[25] Mertz D.P. (1982). [Hallux rigidus arthrosis and gout]. Fortschr. Med..

[26] Bevis M., Marshall M., Rathod T., Roddy E. (2016). The association between gout and radiographic hand, knee and foot osteoarthritis: a cross-sectional study. BMC Muscoskelet. Disord..

[27] Mertz D.P., Mertz A. (1981). [Between hallux rigidus arthrosis and gout is no causal relationship]. Med. Klin..

[28] Bremner J.M., Lawrence J.S., Miall W.E. (1968). Degenerative joint disease in a Jamaican rural population. Ann. Rheum. Dis..

[29] Solomon L., Beighton P., Lawrence J.S. (1976). Osteoarthrosis in a rural South African Negro population. Ann. Rheum. Dis..

[30] Shelton T.J., Singh S., Bent Robinson E., Nardo L., Escobedo E., Jackson L. (2019). The influence of percentage weight-bearing on foot radiographs. Foot Ankle Spec..

[31] Beeson P., Phillips C., Corr S., Ribbans W. (2008). Classification systems for hallux rigidus: a review of the literature. Foot Ankle Int..

[32] Dillard S., Schilero C., Chiang S., Pham P. (2018). Intra- and interobserver reliability of three classification systems for Hallux Rigidus. J. Am. Podiatr. Med. Assoc..

[33] Kubota K., Kamijima Y., Sato T., Ooba N., Koide D., Iizuka H. (2015). Epidemiology of psoriasis and palmoplantar pustulosis: a nationwide study using the Japanese national claims database. BMJ Open.

