# Supplementary material for: Radiographic prevalence and associated factors of hallux rigidus in a large-scale population-based cohort
Source: Osteoarthr Cartil Open. 2025 Oct 11;7(4):100695. doi: 10.1016/j.ocarto.2025.100695 (PMC12589870; doi:10.1016/j.ocarto.2025.100695)
Supplement: Multimedia component 1 [file mmc1.docx]

**Supplementary Table 1.** Comparison of laboratory data and medication use between non-HR and HR groups in males and females

| Variables | | Total | Male | | | | | Female | | | | |
| --- | --- | --- | --- | --- | --- | --- | --- | --- | --- | --- | --- | --- |
|  |  | (N = 1998) | non-HR | HR | MD/OR | 95%CI | P value | non-HR | HR | MD/OR | 95%CI | P value |
|  |  |  | (N = 490) | (N= 164) |  |  |  | (N = 1038) | (N = 206) |  |  |  |
| Laboratory test | |  |  |  |  |  |  |  |  |  |  |  |
|  | UA (mg/dL) | 4.94 (1.29) | 5.79 (1.23) | 5.77 (1.34) | -0.02 | -0.24 to 0.20 | 0.99 | 4.51 (1.10) | 4.56 (1.06) | 0.06 | -0.08 to 0.20 | 0.29 |
|  | LDL (mg/dL) | 124.7 (33.6) | 119.1 (33.4) | 114.9 (32.4) | -4.16 | -10.04 to 1.72 | 0.13 | 127.9 (33.6) | 128.2 (32.3) | 0.30 | -3.95 to 4.56 | 0.99 |
|  | HDL (mg/dL) | 66.9 (17.5) | 59.6 (16.8) | 58.7 (16.2) | -0.88 | -3.83 to 2.07 | 0.52 | 71.1 (16.9) | 68.7 (16.0) | -2.46 | -4.60 to -0.32 | 0.04* |
|  | TG (mg/dL) | 148.9 (101.5) | 176.1 (144.0) | 165.8 (105.5) | -10.28 | -34.27 to 13.71 | 0.76 | 133.8 (75.4) | 147.3 (82.6) | 13.52 | 3.67 to 23.36 | 0.01* |
|  | T. chol (mg/dL) | 215.5 (38.0) | 204.6 (36.0) | 198.7 (35.9) | -5.89 | -12.26 to 0.48 | 0.06 | 221.7 (37.5) | 220.7 (37.4) | -0.97 | -5.76 to 3.82 | 0.68 |
|  | Cre (mg/dL) | 0.758 (0.379) | 0.94 (0.58) | 0.93 (0.24) | -0.01 | -0.10 to 0.08 | 0.44 | 0.67 (0.28) | 0.67 (0.12) | -0.01 | -0.04 to 0.03 | 0.44 |
|  | HbA1c (%) | 5.69 (0.60) | 5.75 (0.70) | 5.72 (0.58) | -0.23 | -0.14 to 0.10 | 0.70 | 5.65 (0.55) | 5.76 (0.58) | 0.12 | 0.05 to 0.19 | <0.001* |
| Medication use | |  |  |  |  |  |  |  |  |  |  |  |
|  | Antihypertensive drugs | 740 (37.4%) | 194 (40.0%) | 72 (44.2%) | 1.19 | 0.83 to 1.70 | 0.35 | 342 (33.3%) | 132 (43.3%) | 1.53 | 1.18 to 1.98 | 0.001* |
|  | Cholesterol-lowering drugs | 571 (28.8%) | 113 (23.3%) | 39 (24.1%) | 1.04 | 0.69 to 1.58 | 0.84 | 292 (28.2%) | 127 (41.9%) | 1.83 | 1.41 to 2.39 | <0.001* |
|  | Triglyceride-lowering drugs | 94 (4.8%) | 29 (6.0%) | 15 (9.3%) | 1.61 | 0.84 to3.08 | 0.15 | 41 (4.0%) | 9 (3.0%) | 0.73 | 0.35 to 1.53 | 0.41 |
|  | Antidiabetic drugs | 177 (9.0%) | 67 (13.8%) | 25 (15.4%) | 1.14 | 0.69 to 1.87 | 0.62 | 57 (5.6%) | 28 (9.2%) | 1.73 | 1.08 to 2.77 | 0.02* |
|  | Urate-lowering drugs | 132 (6.7%) | 72 (14.9%) | 39 (24.1%) | 1.82 | 1.17 to 2.82 | 0.007* | 16 (1.6%) | 5 (1.7%) | 1.06 | 0.38 to 2.92 | >0.99 |
|  | Corticosteroids | 33 (1.7%) | 2 (0.4%) | 2 (1.2%) | 3.03 | 0.42 to 21.70 | 0.26 | 26 (2.5%) | 3 (1.0%) | 0.39 | 0.12 to 1.28 | 0.11 |
|  | Hormonal contraceptives | 13 (0.7%) | 0 (0.0%) | 0 (0.0%) | - | - | - | 10 (1.0%) | 3 (1.0%) | 1.02 | 0.28 to 3.71 | >0.99 |
|  | Antidepressants | 61 (3.1%) | 12 (2.5%) | 4 (2.5%) | 0.99 | 0.32 to 3.13 | >0.99 | 32 (3.1%) | 13 (4.3%) | 1.39 | 0.72 to 2.69 | 0.32 |
|  | Anxiolytics | 322 (16.3%) | 53 (11.0%) | 26 (16.1%) | 1.55 | 0.94 to 2.58 | 0.09 | 181 (17.6%) | 62 (20.4%) | 1.20 | 0.87 to 1.66 | 0.27 |
|  | NSAIDs | 356 (18.0%) | 57 (11.8%) | 31 (19.1%) | 1.77 | 1.10 to 2.86 | 0.02* | 203 (19.8%) | 65 (21.4%) | 1.10 | 0.80 to 1.50 | 0.56 |
|  | Calcium supplements | 75 (3.8%) | 4 (0.8%) | 1 (0.6%) | 0.74 | 0.08 to 6.70 | >0.99 | 56 (5.5%) | 14 (4.7%) | 0.84 | 0.46 to 1.53 | 0.57 |
|  | Vitamin D | 133 (6.8%) | 6 (1.2%) | 2 (1.2%) | 1.00 | 0.20 to 5.02 | >0.99 | 90 (8.8%) | 35 (11.6%) | 1.36 | 0.90 to 2.06 | 0.14 |
|  | Vitamin K | 6 (0.3%) | 0 (0.0%) | 0 (0.0%) | - | - | - | 4 (0.4%) | 2 (0.7%) | 1.69 | 0.31 to 9.27 | 0.63 |
|  | Calcitonin | 10 (0.5%) | 0 (0.0%) | 0 (0.0%) | - | - | - | 9 (0.9%) | 1 (0.3%) | 0.37 | 0.05 to 2.96 | 0.47 |
|  | PTH analogs | 16 (0.8%) | 0 (0.0%) | 1 (0.6%) | - | - | 0.25 | 13 (1.3%) | 2 (0.7%) | 0.52 | 0.12 to 2.32 | 0.54 |
|  | SERMs | 48 (2.4%) | 0 (0.0%) | 0 (0.0%) | - | - | - | 38 (3.7%) | 10 (3.3%) | 0.88 | 0.43 to 1.79 | 0.73 |
|  | Bisphosphonates | 58 (3.0%) | 4 (0.8%) | 0 (0.0%) | - | - | 0.58 | 40 (3.9%) | 14 (4.6%) | 1.19 | 0.64 to 2.21 | 0.59 |
|  | Denosumab | 20 (1.0%) | 1 (0.2%) | 0 (0.0%) | - | - | >0.99 | 17 (1.7%) | 2 (0.7%) | 0.39 | 0.09 to 1.71 | 0.27 |
|  | Antirheumatic | 7 (0.4%) | 0 (0.0%) | 0 (0.0%) | - | - | - | 7 (0.7%) | 0 (0.0%) | - | - | 0.36 |

Abbreviations: UA, uric acid; LDL, low-density lipoprotein cholesterol; HDL, high-density lipoprotein cholesterol; TG, triglycerides; T. chol, total cholesterol; Cre, creatinine; HbA1c, hemoglobin A1c; NSAIDs, nonsteroidal anti-inflammatory drugs; PTH analogs, parathyroid hormone analogs; SERMs, selective estrogen receptor modulators; HR, hallux rigidus; MD, mean difference; OR, odds ratio; CI, confidence interval

Percentages were calculated using the number of participants with available data for each variable as the denominator. The total N varied slightly across variables due to missing responses. Effect measures are presented as MDs with 95% CIs for continuous variables, ORs with 95% CIs for categorical variables.

* Statistically significant difference between non-HR and HR groups, as determined by the chi-square test or Fisher’s exact test for categorical variables, and the Mann–Whitney U test for continuous variables.
